# UCP2 and PRMT1 are key prognostic markers for lung carcinoma patients

**DOI:** 10.18632/oncotarget.20571

**Published:** 2017-08-28

**Authors:** Corina T. Madreiter-Sokolowski, Balázs Győrffy, Christiane Klec, Armin A. Sokolowski, Rene Rost, Markus Waldeck-Weiermair, Roland Malli, Wolfgang F Graier

**Affiliations:** ^1^ Institute of Molecular Biology and Biochemistry, Medical University of Graz, Graz, Austria; ^2^ Dentistry and Maxillofacial Surgery, Medical University of Graz, Graz, Austria; ^3^ MTA TTK Lendület Cancer Biomarker Research Group, Institute of Enzymology, Budapest, Hungary; ^4^ 2nd Department of Pediatrics, Semmelweis University, Budapest, Hungary

**Keywords:** lung cancer cell proliferation, mitochondria, Ca^2+^ controls respiration, protein arginine methyltransferase 1, uncoupling protein 2

## Abstract

Cancer cells have developed unique strategies to meet their high energy demand. Therefore, they have established a setting of Ca^2+^-triggered high mitochondrial activity. But mitochondrial Ca^2+^ uptake has to be strictly controlled to avoid mitochondrial Ca^2+^ overload that would cause apoptotic cell death. Methylation by protein arginine methyl transferase 1 (PRMT1) desensitizes the mitochondrial Ca^2+^ uptake machinery and reduces mitochondrial Ca^2+^ accumulation in cancer cells. In case of PRMT1-driven methylation, proper mitochondrial Ca^2+^ uptake is reestablished by increased activity of uncoupling protein 2 (UCP2), pointing to an importance of these proteins for cancer cell survival and activity. Accordingly, in this study we investigated the impact of UCP2 and PRMT1 on the fate of human lung cancer cells (A549, Calu-3 and H1299) as well as on patients suffering from lung carcinoma. We show that combined overexpression of UCP2 and PRMT1 significantly enhances viability, proliferation as well as mitochondrial respiration. In line with these findings, the overall survival probability of lung carcinoma patients with high mRNA expression levels of UCP2 and PRMT1 is strongly reduced. Furthermore, analysis via The Cancer Genome Atlas (TCGA) reveals upregulation of both proteins, UCP2 and PRMT1, as common feature of various cancer types. These findings suggest that proper mitochondrial Ca^2+^ uptake is essential for devastating tumor growth, and highlight the importance of a tightly controlled mitochondrial Ca^2+^ uptake to ensure proper ATP biosynthesis while avoiding dangerous mitochondrial Ca^2+^ overload. By that, the study unveils proteins of the mitochondrial Ca^2+^ uptake as potential targets for cancer treatment.

## INTRODUCTION

Although more than 100 distinct types of cancer are known, cancer cells share similar molecular characteristics, including loss of cell cycle control, resulting in nearly unlimited replication potential, tissue invasion and metastases [1]. Uncontrolled proliferation of cancer cells comes along with an increased energy demand [2]. For a long time, glycolysis was assumed to be the main source of energy in cancer cells [3]. However, a less active form of pyruvate kinase isoenzyme M2 (PKM2) seems to be the bottleneck in glycolysis of cancer cells [4]. By the reduced conversion of phosphoenolpyruvate to pyruvate, less active PKM2 limits the production of adenosine triphosphate (ATP) from glycolysis, but boosts the accumulation of intermediate products like nucleic acids, phospholipids and serines as essential “building blocks” for cancer cells [4]. Cancer cells with less active PKM2 have to rely on ATP production via oxidative phosphorylation (OXPHOS) in mitochondria. This non-glycolytic metabolic pathway for energy supply is fueled by amino acids as well as by fatty acids that are entering the citrate cycle and firing mitochondrial respiration [4]. Notably, mitochondria's highly efficient ATP production is outranging anaerobic glycolysis and is especially attractive for cells with elevated energy demands [5]. The activity of the mitochondrial respiration chain is strongly reliant on Ca^2+^-dependent dehydrogenases of the citrate cycle in the mitochondrial matrix [6]. In line with that, constitutive Ca^2+^ flux from the ER to mitochondria was demonstrated to be essential to maintain viability of cancer cells with high proliferation activity [7]. The importance of the ER-mitochondrial Ca^2+^ transfer in cancer cells was further highlighted by the finding that several tumor suppressors, including p53, manipulate contact sites between these two organelles in regions of mitochondria-associated-ER membranes (MAMs), where ER and mitochondria are in close proximity to each other to ensure Ca^2+^ and lipid flux [8, 9]. Recently, we showed that common cancer cell lines exhibit a stronger tethering between mitochondria and ER in comparison to non-cancerous cells, which makes them more vulnerable for mitochondrial Ca^2+^ overload, induced by, for instance, the polyphenol resveratrol [10]. However, cancer cells seem to have a unique strategy to protect themselves against lethal mitochondrial Ca^2+^ accumulation. Methylation of the mitochondrial Ca^2+^ uptake 1 (MICU1) [11], a protein controlling the activity of the mitochondrial Ca^2+^ uniporter (MCU) [12], by protein arginine methyl transferase 1 (PRMT1) causes a strong sensitivity loss of the mitochondrial Ca^2+^ uptake machinery in cancer cells and counteracts the risk of mitochondrial Ca^2+^ overload [13]. Nevertheless, to ensure sufficient mitochondrial Ca^2+^ uptake, the uncoupling protein 2 (UCP2) is able to normalize mitochondrial Ca^2+^ uptake in case of PRMT1-driven methylation of MICU1 in cancer cells [13]. Accordingly, we hypothesize that cancer cells develop an exclusive strategy for balancing mitochondrial Ca^2+^ uptake through the expression pattern of UCP2 and PRMT1 in order to meet the Ca^2+^ demand of the dehydrogenases to fuel OXPHOS, while protecting themselves from lethal mitochondrial Ca^2+^ accumulation. In line with our hypothesis, recent studies have already linked the expression of either UCP2 or PRMT1 to tumor growth, state of metastasis and chemo resistance [14–16]. In this study, we considered the recent reports on the functional interaction of UCP2 and PRMT1 [13] and investigated the importance of this relation on cancer cell's viability, proliferation and patient's survival.

## RESULTS

### Combined overexpression of UCP2 and PRMT1 increases cell viability and proliferation of human lung cancer cells

**Figure 1:**
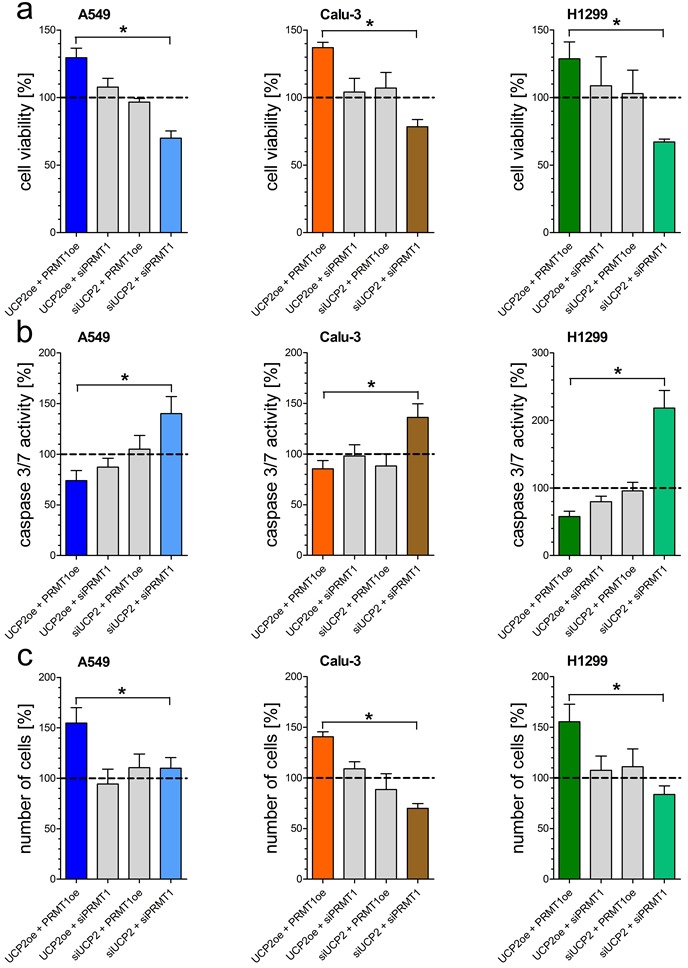
Impact of UCP2 and PRMT1 on cell viability, caspase activity and proliferation of lung carcinoma cell lines Cell viability of A549 *(left panel)*, Calu-3 *(middle panel)* and H1299 *(right panel)* cells with knockdown of UCP2 or PRMT1 and/or overexpression of UCP2 and PRMT1 was measured via Celltiter-Blue assay and calculated as percentage of viable cells normalized to control condition **a.** Caspase activity of A549 *(left panel)*, Calu-3 *(middle panel)* and H1299 *(right panel)* cells, treated with siRNA against UCP2 or PRMT1 and/or overexpressing UCP2 and PRMT1, was measured by Caspase 3/7-Glo assay and normalized to control conditions **b.** Cellular proliferation of A549 *(left panel)*, Calu-3 *(middle panel)* and H1299 *(right panel)* cells diminished of UCP2 and PRMT1 and/or overexpressing these proteins was determined by counting cells 48 h after seeding and cell number was normalized to control condition **c.** Bar graphs represent mean +/- SEM *(n=3)*.

Since mitochondrial Ca^2+^ uptake is essential for mitochondrial metabolic activity, we manipulated the expression of PRMT1 and UCP2, proteins known to affect the mitochondrial Ca^2+^ uptake machinery, to investigate their effect on human non-small-cell lung cancer cell lines A549, Calu-3 and H1299. Origin of each cell lines was validated by Sanger sequencing [Supplementary Figure 1A]. Knockdown or overexpression, verified by qRT-PCR [Supplementary Figure 1B], of either UCP2 or PRMT1 alone did not affect cell viability and apoptotic caspase 3/7 activity. Combined depletion of UCP2 and PRMT1 by specific siRNAs strongly decreased cell viability [Figure 1A], whereas activity of apoptotic caspases 3/7 was significantly increased [Figure 1B]. In contrast, overexpression of both proteins improved cell viability and diminished apoptotic caspase 3/7 activity. In line with these data, cancer cell proliferation was significantly enhanced by combined overexpression of UCP2 and PRMT1 in comparison to cells with double knockdown [Figure 1C].

### Combined overexpression of UCP2 and PRMT1 results in enhanced mitochondrial respiration activity

**Figure 2:**
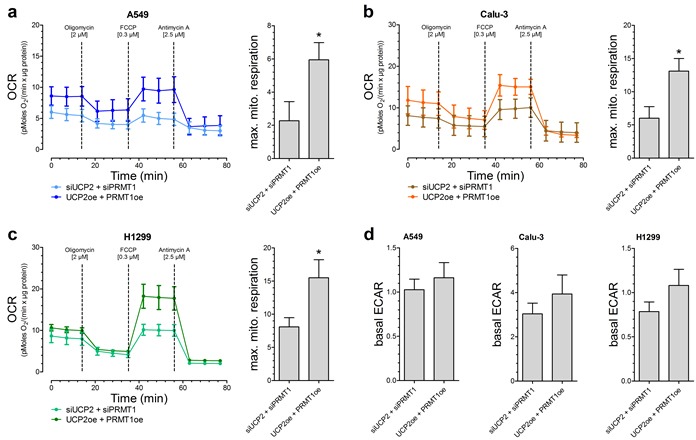
Mitochondrial respiration and basal rate of glycolysis in different lung carcinoma cell lines Oxygen consumption rate (OCR) of A549 *(n=6)* **a.**, Calu-3 *(n=5)* **b.** and H1299 *(n=6)* **c.** cells with either combined overexpression or knockdown of UCP2 and PRMT1. 2 μM Oligomycin, 0.3 μM FCCP and 2.5 μM Antimycin A were injected as indicated. Bar graphs (mean +/- SEM) represent maximal mitochondrial respiration (max. mito. respiration) of the different cells. Rate of basal extracellular acidification (ECAR) of A549 *(left panel, n=6)*, Calu-3 *(middle panel, n=5)* and H1299 *(right panel, n=6)* cells was presented as bar graphs (mean +/- SEM) **d.**

Based on our previous work [13], we assume that this positive impact of UCP2 and PRMT1 on cancer cell viability and proliferation is due to a more efficient mitochondrial respiration. Therefore, OCR was determined after addition of metabolic modulators. Indeed, A549 [Figure 2A], Calu-3 [Figure 2B] as well as H1299 [Figure 2C] cells with combined overexpression of UCP2 and PRMT1 had an enhanced maximal mitochondrial respiration, representing an increased capacity to create ATP via mitochondrial respiration, in comparison to cells with transient knockdown of UCP2 and PRMT1, whereas the basal rate of glycolysis (ECAR) was not altered [Figure 2D].

### Survival rate of lung carcinoma patients is influenced by expression of UCP2 and PRMT1

**Figure 3:**
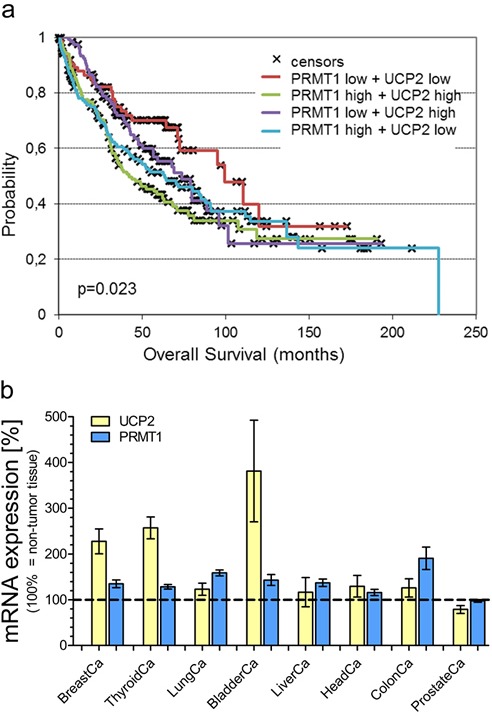
Survival analysis of lung carcinoma patients and mRNA expression analysis Overall survival probability of lung carcinoma patients was analyzed in regard to the individual mRNA expression of UCP2 and PRMT1 (UCP2-high/PRMT1-high vs. UCP2-high/PRMT1-low vs. UCP2-low/PRMT1-high vs. UCP2-low/PRMT1-low) and presented as Kaplan-Meier survival plot **a.** mRNA expression levels of UCP2 and PRMT1 in different cancer types were determined via TCGA analysis and presented as bar graphs, normalized to mRNA expression levels of UCP2 and PRMT1 in adjacent normal tissue (BreastCa/breast invasive carcinoma: *n=114*; ThyroidCa/thyroid carcinoma: *n=59*; LungCa/lung carcinoma: *n=109*; BladderCa/bladder urothelial carcinoma: *n=19*; LiverCa/liver hepatocellular carcinoma: *n=50*; HeadCa/head and neck squamous cell carcinoma: *n=43*; ColonCa/colorectal adenocarcinoma: *n=32*; ProstateCa/prostate cancer: *n=*52) **b.**

Since viability as well as proliferation of lung cancer cells were strongly affected by expression levels of UCP2 and PRMT1, we analyzed the survival of lung carcinoma patients with different mRNA expression patterns of UCP2 and PRMT1. The median value of mRNA expression was used as cutoff value and the patient group was divided into four cohorts (PRMT1-high/UCP2-high; PRMT1-low/UCP2-low; PRMT1-high/UCP2-low; PRMT1-low/UCP2-high), whose overall survival time was analyzed. The overall survival probability [Figure 3A] was strongly decreased in the cohort with mRNA expression levels of both, PRMT1 and UCP2, above the median value in comparison to the group with low expression levels of both genes (p = 0.023). High mRNA expression of one of these genes already negatively affected overall survival (PRMT1-high/UCP2-low vs. PRMT1-low/UCP2-low: p=0.0406; PRMT1-low/UCP2-high vs. PRMT1-low/UCP2-low: p=0.16), but not in such an extent as combined high mRNA expression of PRMT1 and UCP2. In particular, 43% of patients with simultaneously high PRMT1 and UCP2 expression, while 70% of patients with simultaneously low PRMT1 and UCP2 expression were alive at 5 years follow-up.

### Combined upregulation of UCP2 and PRMT1 as a common feature of numerous cancer types

Our results from cell viability experiments with human lung carcinoma cells as well as analysis of lung carcinoma patient's overall survival reveal a positive impact of UCP2 and PRMT1 on cancer cells. Therefore, we investigated whether high mRNA expression levels of UCP2 and PRMT1 are a common feature in cancerous tissues. Analysis of The Cancer Genome Atlas (TCGA) database revealed an increase in UCP2 as well as in PRMT1 mRNA expression in several tumor tissues in comparison to adjacent normal tissue [Figure 3B]. Only prostate adenocarcinoma was found to have low levels of PRMT1 mRNA expression. Notably, in this particular type of cancer also UCP2 expression was lower than in the adjacent normal tissue, possibly demonstrating that the mRNA expression of UCP2 and PRMT1 is dependent on the respective other protein [Figure 3B].

## DISCUSSION

Recently, we have shown that PRMT1-driven methylation of MICU1 desensitizes the mitochondrial Ca^2+^ uptake machinery and, therefore, reduces mitochondrial Ca^2+^ accumulation in cancer cells [13]. Hence, under these conditions proper mitochondrial Ca^2+^ uptake is reestablished by UCP2 that normalizes the sensitivity of MICU1 and, thus, reestablishes normal mitochondrial Ca^2+^ uptake [13]. Since an increased tethering between mitochondria and the biggest internal Ca^2+^ store, the ER, was found in cancer cells [10], we hypothesize that cancer cells with high levels of PRMT1-driven methylation control their mitochondrial Ca^2+^ uptake via UCP2 to ensure suitable mitochondrial ATP production by stimulating dehydrogenases with a proper amount of Ca^2+^. Elevated levels of mitochondrial respiration in lung carcinoma cells with combined overexpression of UCP2 and PRMT1 are in line with this suggestion. The finding that viability and proliferation of lung carcinoma cells was strongly increased in case of upregulated PRMT1 and UCP2 expression further supports a link between the expression of these two proteins and cancer cells’ perilousness. In line with this assumption, cancer cells depleted from PRMT1 and UCP2 showed an increased vulnerability and exhibited a largely decreased cell viability and proliferation. The remarkably high levels of apoptotic caspase activity in these cells are likely a result of PRMT1-uncontrolled mitochondrial Ca^2+^ uptake and cell death by mitochondrial Ca^2+^ overload due to the enhanced tethering between the ER and mitochondria in these cells [10]. Notably, unaltered cell viability, apoptotic caspase activity and proliferation in cells depleted of either UCP2 or PRMT1, might point to a still functional interrelation between both proteins as recently described [13]. These results obtained from cell culture experiments are in line with our overall survival analyses of patients suffering from lung cancer, which has already been linked to a strongly altered Ca^2+^ homeostasis [17]. In particular, the negative correlation of survival probability of patients with the expression of UCP2 and PRMT1 supports our hypothesis about the impact of proper mitochondrial Ca^2+^ uptake on the metabolic efficiency of cancer cells that affects cancer abrasiveness and patient survival. On the other hand, the present data suggest that cancer cells in the patient cohort with low levels of UCP2 and PRMT1 might be metabolically less active, yielding attenuated cancer cell proliferation and, thus, increased patient survival. The overall survival of patients with high mRNA expression levels of PRMT1 alone was already significantly reduced in comparison to those patients with low mRNA expression levels of PRMT1 and UCP2. Therefore, we conclude that protection from mitochondrial Ca^2+^ overload by a PRMT1-controlled mitochondrial Ca^2+^ uptake is already a major advantage for cancer cells. Improper mitochondrial Ca^2+^ accumulation as Achilles’ heel of cancer cells has been already reported: *i*, in prostate cancer cells, mitochondrial Ca^2+^ overload was proven to strongly enhance apoptosis, induced by TRAIL (TNFα related apoptosis inducing ligand) [18]. Notably, prostate cancer was the only cancer type found in our TCGA analysis with low levels of PRMT1, which makes this specific cancer probably more vulnerable for mitochondrial Ca^2+^ overload. *ii*, the expression levels of MICU1 was shown to correlate with the overall survival of ovarian cancer patients as well as cancer cell chemoresistance against cisplatin [19]. *iii*, in triple-negative breast cancer, the most aggressive type of breast cancer, upregulation of the channel forming protein responsible for mitochondrial Ca^2+^ uptake, MCU, was demonstrated to positively correlate with tumor size and lymph node infiltration [20]. One might argue, that overexpression of MCU might lead to mitochondrial Ca^2+^ overload and apoptosis as in prostate cancer cells, but, breast cancer is well known for a strong upregulation of PRMT1 [21], also confirmed by our TCGA analysis, which might protect cancer cells from Ca^2+^ overload via MICU1 methylation. *iv*, strongly elevated UCP2 levels have been linked to poorly differentiated breast cancer, indicating that similar as MCU also UCP2 helps breast cancer cells to proliferate, migrate and invade [14]. Therefore, a combined upregulation of PRMT1 and UCP2 as crucial risk in various cancer types, as found in our TCGA analysis, seems to be reasonable.

All together, these findings suggest that proper mitochondrial Ca^2+^ uptake might be essential for devastating tumor growth, and highlight the importance of a tightly-controlled mitochondrial Ca^2+^ uptake to prevent mitochondrial Ca^2+^ overload and, ultimately, cell death. Our study reveals a major role of PRMT1 and UCP2 in cancer cell viability and proliferation as well as in patients’ overall survival probability, indicating that numerous different cancer types utilize upgraded mitochondrial Ca^2+^ uptake to meet their greatly enhanced energy demand. Accordingly, targeting mitochondrial Ca^2+^ uptake proteins by newly developed compounds against distinct members of the mitochondrial Ca^2+^ uptake machinery would allow a personalized therapy that considers individual expression pattern in the very tumor, in order to efficiently evoke cancer cell injury and increased susceptibility against therapeutics.

## MATERIALS AND METHODS

### Cell culture, sanger sequencing and transfection

H1299 cells were grown in Dulbecco's Modified Eagle Medium (DMEM) from Sigma Aldrich (Vienna, Austria), A549 and Calu-3 cells in a 1:1 mixture of Ham‘s F12 and DMEM. Media were supplemented with 10% fetal bovine serum, 100 U/ml penicillin, 100 μg/ml streptomycin and 1.25 μg/ml amphotericin B (Gibco, Lifetechnologies; Vienna, Austria). To verify the origin of the various cell lines, cell lysates were sent to Microsynth (Balgach, Switzerland) for Sanger sequencing. Cells were transiently transfected at a confluence of 60 - 80% with 1.5 μg plasmid DNA encoding UCP2 or PRMT1 as well as with 100 μM siRNA UCP2 or PRMT1 using 2.5 μl of TransFast^TM^ transfection reagent (Promega; Madison, WI, US) in 1 ml of serum- and antibiotic-free medium. Transfection mix was replaced by full culture medium after 24 hours. All experiments were performed 48 hours after transfection. siRNAs were obtained from Microsynth, and their sequences (5′-3′) were as follows: UCP2

5′-CACTGTCGACGCCTACAAGACCATC-3′, ‘5′-GTCATAGGTCACCAGCTCAGCACAG-3′; PRMT1: 5′-TGCTCAACACCGTGCTCTATGC-3′, 5′-TCCTCGATGGCCGTCACATACA-3′.

### mRNA isolation and qRT-PCR

Total RNA from cells was isolated using the PEQLAB total RNA isolation kit (Erlangen, Germany) and reverse transcription was performed using a cDNA synthesis kit (Applied Biosystems; Foster City, CA). qRT-PCR was performed using QuantiFast SYBR Green RT-PCR kit (Qiagen; Hilden, Germany) as described previously [13]. Primers for qRT-PCR were obtained from Invitrogen (Vienna, Austria), and their sequences (5′-3′) were: UCP2 forward, TCCTGAAAGCCAACCTCATG; UCP2 reverse, GGCAGAGTTCATGTATCTCGTC; PRMT1 forward, TGCTCAACACCGTGCTTATGC; PRMT1 reverse, TCCTCGATGGCCGTCACATACA

### Cell viability and apoptosis assay

For cell viability and apoptosis assays, cells were plated 24 h after transfection on 96-well plates at a density of 5,000 cells/well. Cell viability was measured 48 h after transfection using CellTiter-Blue assay and apoptotic caspase activity was determined by using the Caspase-Glo^®^ 3/7 assay (Promega; Madison, WI, US) as reported previously [10].

### Proliferation assays

For proliferation determination, cells were plated 24 h after transfection on 6-well plates at a density of 8,000 cells/well. Number of cells was counted 48 h after transfection and presented as percentage of control cell proliferation.

### Measurement of mitochondrial respiration

24 h after transfection, A546, Calu-3 and H1299 cells were plated on Cell-Tak™ coated XF96 polystyrene cell culture microplates (Seahorse Bioscience^®^, Agilent; California, US) at a density of 50,000 cells per well. After 24 h cells were washed and pre-incubated in XF assay medium supplemented with 1 mM sodium pyruvate, 2 mM glutamine and 5.5 mM D-glucose. Oxygen consumption rate (OCR) and extracellular acidification rate (ECAR) were subsequently measured every 7 min using an XF96 extracellular flux analyzer. After 15 min basal measurement, Oligomycin [2 μM], FCCP [0.3 μM] and Antimycin A [2.5 μM] were injected. Oxygen consumption was normalized to protein content (pmol O_2_/(min×μg protein)).

### Clinical data acquisition

According to mRNA expression of PRMT1 and UCP2, Kaplan-Meier survival plots presenting the survival of lung carcinoma patients were calculated using data from Gene Expression Omnibus (GEO). Patient samples were split into four groups regarding expression of the indicated genes (median as cutoff value). These four patient cohorts were compared by a Kaplan-Meier survival plot and logrank P values were calculated.

### Data acquisition via Xena

The mRNA expression levels of the genes coding for PRMT1 and UCP2 in various tumor tissues were obtained as RNA SeqV2 RSEM values through UCSC Xena (https://xenabrowser.net/) as The Cancer Genome Atlas (Provisional, TCGA) datasets in April 2017. The selected genomic profile was ‘gene expression RNAseq (polyA+ IlluminaHiSeq)’ and the entered gene set was a user-defined list. Expression levels of normal and tumor tissue samples with the same sample ID were matched [22]. The mRNA expression levels of tumor tissues were normalized to the mRNA expression levels of corresponding adjacent healthy tissue samples from the same patient (100% value). Patients with missing expression levels of normal or tumor tissues were excluded.

### Statistical analysis

The statistical analysis was performed with GraphPad Prism 5.0 using the unpaired Student's t-test, and p<0.05 was considered to be significant.

## SUPPLEMENTARY FIGURE



## Abbreviations

(ATP): adenosine triphosphate
(DMEM): Dulbecco's Modified Eagle Medium
(ECAR): extracellular acidification rate
(GEO): Gene Expression Omnibus
(MAMs): mitochondria-associated-ER membranes
(MCU): mitochondrial Ca^2+^ uniporter
(MICU1): mitochondrial Ca^2+^ uptake 1
(OXPHOS): oxidative phosphorylation
(OCR): oxygen consumption rate
(PRMT1): protein arginine methyl transferase 1
(PKM2): pyruvate kinase isoenzyme M2
(TCGA): The Cancer Genome Atlas
(UCP2): uncoupling protein 2
