# Supplementary material for: UCP2 and PRMT1 are key prognostic markers for lung carcinoma patients
Source: Oncotarget. 2017 Aug 28;8(46):80278–85. doi: 10.18632/oncotarget.20571 (PMC5655196; doi:10.18632/oncotarget.20571)
Supplement: Supplementary file 1 [file oncotarget-08-80278-s001.pdf]

UCP2 and PRMT1 are key prognostic markers for lung carcinoma patients

SUPPLEMENTARY MATERIALS

1a

Analysis Results for Customer Sample A549

1. Summary Table of the STR Profile

Locus	Chromosomal Location	ATCC Marker	Customer Sample Typed Alleles	Database Alleles	Comments
D3S1358	Chr03		16	N/A	
TH01	Chr11	Yes	8/9.3	8/9.3	
D21S11	Chr21		29	N/A	
D18S51	Chr18		14/17	N/A	
Penta_E	Chr15		7/11	N/A	
D5S818	Chr05	Yes	11	11	
D13S317	Chr13	Yes	11	11	
D7S820	Chr07	Yes	8/11	8/11	
D16S539	Chr16	Yes	11/12	11/12	
CSF1PO	Chr05	Yes	10/12	10/12	
Penta_D	Chr21		9	N/A	
AMEL	X/Y	Yes	X	X/Y	
vWA	Chr12	Yes	14	14	
D8S1179	Chr08		13/14	N/A	
TPOX	Chr2	Yes	8/11	8/11	
FGA	Chr04		23	N/A	

2. Electropherogram

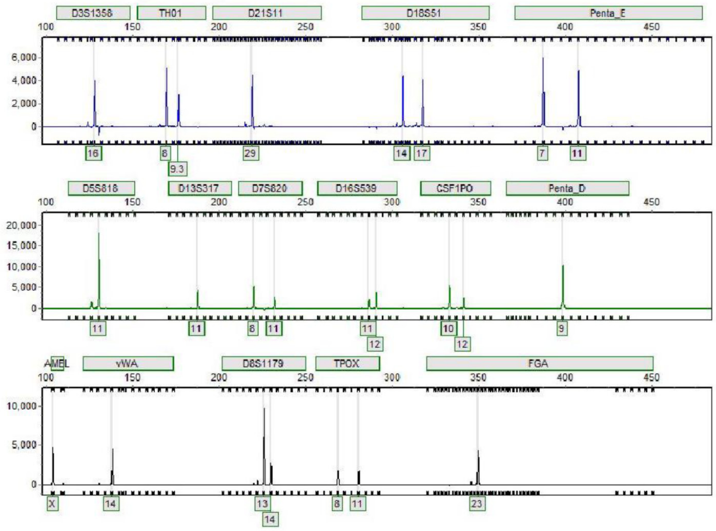

3. Conclusion

According to our analysis of the submitted sample there is no contamination with human origin.

The analyzed data of the submitted sample match 100 % to the DNA profile of the cell line A549 (ATCC® CRM-CCL-185™) and 100 % over all 15 autosomal STRs to the Microsynth's DNA reference profile of A549.

Analysis Results for Customer Sample Calu-3

1. Summary Table of the STR Profile

Locus	*Chromosomal Location*	*ATCC Marker*	*Customer Sample Typed Alleles*	*Database Alleles*	*Comments*
D3S1358	Chr03		15/18	N/A	
TH01	Chr11	Yes	6/9.3	6/9.3	
D21S11	Chr21		28/30	N/A	
D18S51	Chr18		14/17	N/A	
Penta_E	Chr15		5/21	N/A	
D5S818	Chr05	Yes	11	11	
D13S317	Chr13	Yes	12	12	
D7S820	Chr07	Yes	10/11	10/11	
D16S539	Chr16	Yes	12/14	12/14	
CSF1PO	Chr05	Yes	11/12	11/12	
Penta_D	Chr21		9/16	N/A	
AMEL	X/Y	Yes	X	X	
vWA	Chr12	Yes	16/17	16/17	
D8S1179	Chr08		11/15	N/A	
TPOX	Chr2	Yes	8/12	8	Allel 12: Ratio
FGA	Chr04		25	N/A	

2. Electropherogram

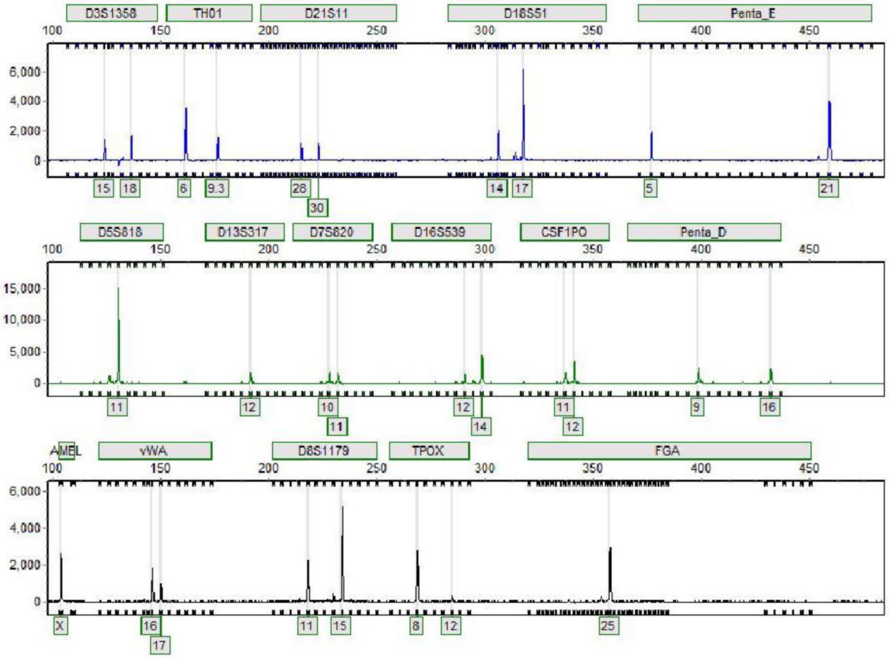

3. Conclusion

According to our analysis of the submitted sample there is no contamination with human origin.

The analyzed data of the submitted sample match 93.8 % to the DNA profile of the cell line Calu-3 (ATCC® HTB-55™) and 96.7 % over all 15 autosomal STRs to the DNA reference profile of Calu-3 (Cellosaurus, RRID:CVCL_0609).
Cell line samples matching at ≥ 80 % of alleles across the eight reference loci are said to be related.

There is an allelic imbalance in the STR TPOX: The peak height ratio is < 25 %.

Analysis Results for Customer Sample NCI-H1299

1. Summary Table of the STR Profile

Locus	Chromosomal Location	ATCC Marker	Customer Sample Typed Alleles	Database Alleles	Comments
D3S1358	Chr03		17	N/A	
TH01	Chr11	Yes	6/9.3	6/9.3	
D21S11	Chr21		32.2	N/A	
D18S51	Chr18		16	N/A	
Penta_E	Chr15		11	N/A	
D5S818	Chr05	Yes	11	11	
D13S317	Chr13	Yes	12	12	
D7S820	Chr07	Yes	10	10	
D16S539	Chr16	Yes	12/13	12/13	
CSF1PO	Chr05	Yes	12	12	
Penta_D	Chr21		13	N/A	
AMEL	X/Y	Yes	X	X	
vWA	Chr12	Yes	16/18	16/17/18	
D8S1179	Chr08		10/13	N/A	
TPOX	Chr2	Yes	8	8	
FGA	Chr04		20	N/A	

2. Electropherogram

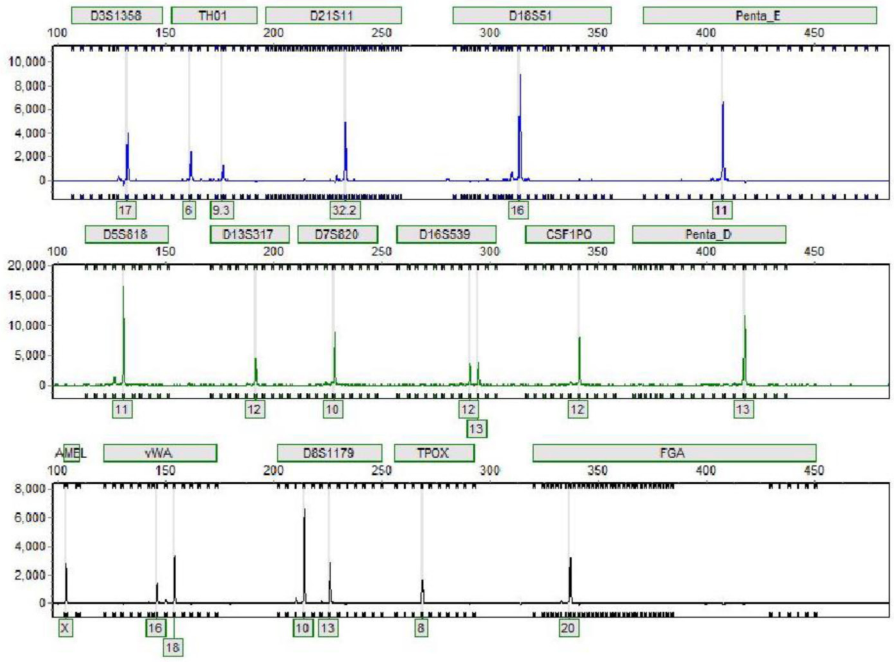

3. Conclusion

According to our analysis of the submitted sample there is no contamination with human origin.

The analyzed data of the submitted sample match 100 % to the DNA profile of the cell line NCI-H1299 (ATCC® CRL-5803™) and 100 % over all 15 autosomal STRs to the Microsynth's DNA reference profile of NCI-H1299.

1b

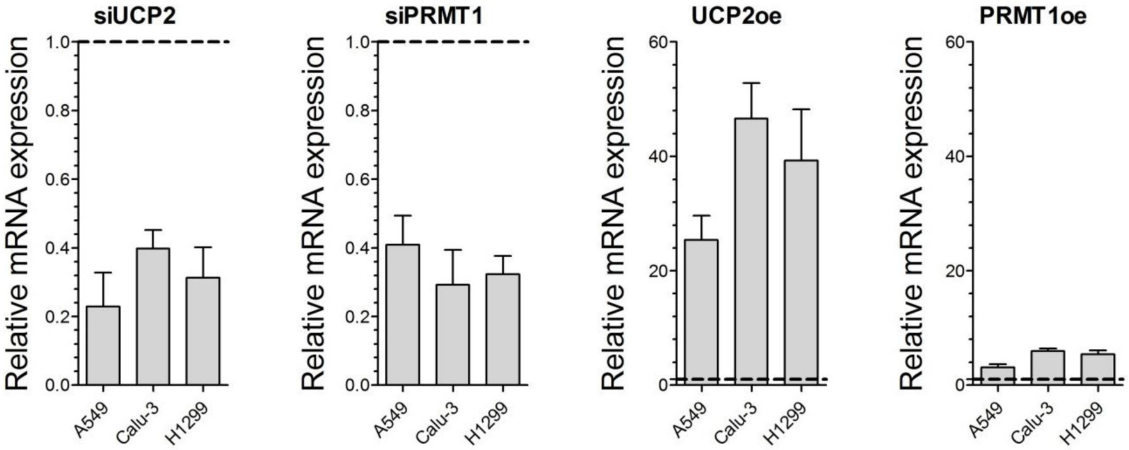

Supplementray Figure 1: Important data on cell origin including sanger sequencing to fullfil international standards
